# The Symbiotic Performance of Chickpea Rhizobia Can Be Improved by Additional Copies of the *clpB* Chaperone Gene

**DOI:** 10.1371/journal.pone.0148221

**Published:** 2016-02-04

**Authors:** Ana Paço, Clarisse Brígido, Ana Alexandre, Pedro F. Mateos, Solange Oliveira

**Affiliations:** 1 ICAAM–Instituto de Ciências Agrárias e Ambientais Mediterrânicas (Laboratório de Microbiologia do Solo), Universidade de Évora, Núcleo da Mitra, Ap. 94, 7002–554, Évora, Portugal; 2 IIFA–Instituto de Investigação e Formação Avançada, Universidade de Évora, Ap. 94, 7002–554, Évora, Portugal; 3 Departamento de Microbiología y Genética, Centro Hispano Luso de Investigaciones Agrarias, Universidad de Salamanca, 37007, Salamanca, Spain; Estacion Experimental del Zaidin—CSIC, SPAIN

## Abstract

The ClpB chaperone is known to be involved in bacterial stress response. Moreover, recent studies suggest that this protein has also a role in the chickpea-rhizobia symbiosis. In order to improve both stress tolerance and symbiotic performance of a chickpea microsymbiont, the *Mesorhizobium mediterraneum* UPM-Ca36^T^ strain was genetically transformed with pPHU231 containing an extra-copy of the *clpB* gene. To investigate if the *clpB*-transformed strain displays an improved stress tolerance, bacterial growth was evaluated under heat and acid stress conditions. In addition, the effect of the extra-copies of the *clpB* gene in the symbiotic performance was evaluated using plant growth assays (hydroponic and pot trials). The *clpB*-transformed strain is more tolerant to heat shock than the strain transformed with pPHU231, supporting the involvement of ClpB in rhizobia heat shock tolerance. Both plant growth assays showed that ClpB has an important role in chickpea-rhizobia symbiosis. The nodulation kinetics analysis showed a higher rate of nodule appearance with the *clpB*-transformed strain. This strain also induced a greater number of nodules and, more notably, its symbiotic effectiveness increased ~60% at pH5 and 83% at pH7, compared to the wild-type strain. Furthermore, a higher frequency of root hair curling was also observed in plants inoculated with the *clpB*-transformed strain, compared to the wild-type strain. The superior root hair curling induction, nodulation ability and symbiotic effectiveness of the *clpB*-transformed strain may be explained by an increased expression of symbiosis genes. Indeed, higher transcript levels of the nodulation genes *nodA* and *nodC* (~3 folds) were detected in the *clpB*-transformed strain. The improvement of rhizobia by addition of extra-copies of the *clpB* gene may be a promising strategy to obtain strains with enhanced stress tolerance and symbiotic effectiveness, thus contributing to their success as crop inoculants, particularly under environmental stresses. This is the first report on the successful improvement of a rhizobium with a chaperone gene.

## Introduction

The need of more sustainable agriculture practices, namely the reduction of chemical fertilizers, highlights the importance of biological nitrogen fixation by symbiotic legume-rhizobia associations (e.g. [1, 2]). The establishment of these plant-bacteria symbioses is initiated by a complex signalling dialogue between both partners, allowing the entry of rhizobia into the root and the development of nodules, which are plant organs where rhizobia bacteroids reduce atmospheric nitrogen into ammonia that can be used by the plant [3, 4]. Nodulation and symbiotic N_2_-fixation involve many rhizobial genes, which are commonly designated as symbiosis genes, such as *nod* and *nif* genes [5]. For example, the genes *nodABC* (transcriptionally regulated by NodD) encode enzymes that synthesize Nod factors (lipo-chitoolgosaccharides), which are perceived by the plant, activating the root hair curling, forming a hook that enclose the bacteria and leads to the subsequent development of an infection thread [5]. Nevertheless, previous studies have shown that chaperone proteins, such as GroEL and ClpB, typically involved in stress response, may also play important roles in the symbiotic legume-rhizobia relationships [6–8].

Stressful environmental conditions are limiting factors for the growth and survival of both legume and rhizobia symbiotic partners, mostly by the perturbation of the cellular proteins homeostasis [9, 10]. The nodule formation and nitrogen fixation in bacteroids [11] are highly affected by stress conditions (e.g. [12, 13]). Therefore, it is likely that proteins involved in rhizobia stress response, as chaperone proteins that act to prevent protein aggregation, assist refolding and mediate degradation of misfolded proteins [14], have a role in the symbiotic performance of these bacteria. For instance, alfalfa plants inoculated with *groELc* knockout mutants of *Ensifer meliloti* presented a delay in nodulation compared to plants inoculated with the wild-type strain, and the nodules induced by these mutants show no nitrogen fixation [8]. Similarly, chickpea plants inoculated with a *clpB* knockout mutant of *Mesorizobium ciceri* showed a delay in nodulation and a lower number of bacteroids in the nodules, in comparison with plants inoculated with the wild-type strain [7].

The ClpB chaperone (belonging to the Hsp100 proteins family) is considered an important protein in the response to stress conditions, since it is an ATP-dependent disaggregase, which has the remarkable ability to disaggregate and activate aggregated proteins accumulating under stress conditions (e.g. [15, 16]). Many studies, in prokaryotes and eukaryotes report the primordial role of ClpB or its homologous proteins in thermotolerance [17–20], but its action seems also important for the survival to a variety of other acute stress conditions [21], such as osmotic, ethanol, acidity and salinity stresses (e.g. [22–25]). In bacteria, ClpB is a multidomain protein composed of an N-terminal domain, two ATP-binding domains (AAA+ domains), termed AAA1 and AAA2, and a middle domain inserted into AAA1, the coiled-coil M-domain (e.g. [26, 27]). To achieve its function, ClpB cooperates with other proteins, namely from the DnaKJ system (e.g. [28, 29]). Its specific role in protein disaggregation comprises the extraction of polypeptides from aggregated particles and their translocation through the ClpB central pore hexamers [27, 30, 31].

In an attempt to improve the symbiotic performance of a chickpea *Mesorhizobium*, the type strain *Mesorhizobium mediterraneum* UPM-Ca36^T^ was transformed with extra-copies of the *clpB* gene cloned in the expression vector pPHU231. This strain was selected due to its high sensitivity to different environmental stress conditions [32], as well as low symbiotic effectiveness [33]. The phenotype of the *clpB*-transformed strain was evaluated in free-living conditions and in symbiosis with chickpea, in order to further characterize the role of this chaperone in rhizobia.

## Material and Methods

### Transformation of *M*. *mediterraneum* UPM-Ca36^T^ with extra-copies of the *clpB* gene

The *M*. *mediterraneum* UPM-Ca36^T^ *clpB* gene (GenBank accession number: KT285182, this work), plus its promoter and terminator regions, were amplified by PCR (primers ClpB-BamHI-F 5’-GGATCCCGCCGTTTTTGTTTGTGCGC-3’ and ClpB-BamHI-R 5’-GGATCCATCCATTTCATGCCGCGTGA-3’), generating a fragment of 3059 bp. These primers were designed based on the draft genome of *M*. *mediterraneum* UPM-Ca36^T^ (unpublished data) and include a recognition site for the endonuclease *BamHI*, used to clone in the expression vector pPHU231, a low copy plasmid vector [34]. The identification of the putative promoter and terminator regions was previously performed using BPROM-Prediction of bacterial promoters software (http://www.softberry.com) and ARNold Finding Terminators at IGM—Web Server (http://rna.igmors.u-psud.fr/toolbox/arnold/), respectively. Total DNA was extracted using the E.Z.N.A. bacterial DNA kit (Omega Bio-Tek, Norcross, U.S.A.), according to the manufacturer’s instructions. The PCR reaction was performed in a final volume of 50 μL, using 20 ng of total DNA, 1× reaction buffer, 0.2 mM of each dNTP, 1.5mM of MgSO_4_, 15 pmol of each primer and 0.02 U of KOD Hot Start DNA polymerase (Merck Millipore, Darmstadt, Germany). The amplification program was: 2 min of initial denaturation at 95°C, and 30 cycles of 10 s at 95°C, 10 s at 65°C and 85 s at 70°C. The PCR product was purified using the GFX DNA purification Kit (GE Healthcare, Little Chalfont, UK), cloned in pCR-Blunt^TM^ vector (ThermoFisher Scientific, Waltham, U.S.A), and sequenced. Subsequently, the cloned *clpB* fragment was introduced as a *BamHI* fragment into the expression vector pPHU231, and transformed into *Escherichia coli* DH5α. To ensure that the expression of the cloned *clpB* gene was occurring from its native promoter and not from the *lacZ* promoter, the fragment was cloned with the terminator region downstream of the *lacZ* promoter. The pPHU231 plasmid containing the *clpB* gene (pPHUclpB) was introduced in *M*. *mediterraneum* UPM-Ca36^T^ strain by triparental mating, as described in [35], generating strain Ca36pPHUclpB. All plasmids and bacteria used in this work are presented in Table 1. *M*. *mediterraneum* UPM-Ca36^T^ transformed with the pPHU231 plasmid (Ca36pPHU) was also obtained and used to compare bacteria that differ only in additional copies of the *clpB* gene.

**Table 1:** Bacterial strains and plasmids used in the present work.

Plasmid/Strain	Characteristics	Reference
pRK600	Helper plasmid pRK2013 *npt*::Tn*9*, Cm^r^	[36]
pPHU231	pRK290 derivative, broad-host-range vector, Tc^r^	[34]
pPHUclpB	pPHU231 with a copy of *M*. *mediterraneum* UPM-Ca36^T^ *clpB* gene including its promoter and terminator regions	This work
*E*. *coli*		
MT616	MT607 (pRK600)	[36]
DH5α	*SupE44 Δ lacU169 (φ80lacZΔM15) hsdR17 recA1 endA1 gyrA96 thi-1 relA1*	[37]
*Mesorhizobium*		
Ca36WT	*M*. *mediterraneum* UPM-Ca36^T^	[38]
Ca36pPHU	*M*. *mediterraneum* UPM-Ca36^T^ carrying pPHU231	This work
Ca36pPHUclpB	*M*. *mediterraneum* UPM-Ca36^T^ carrying pPHU231 with a copy of *M*. *mediterraneum* UPM-Ca36^T^ *clpB* gene, including its promoter and terminator regions	This work

The presence of the pPHU231 or pPHUclpB plasmids in *M*. *mediterraneum* UPM-Ca36^T^ was confirmed by PCR, using universal M13 primers. These PCR reactions were carried out in a final volume of 25 μL, using 2.5 μL of total DNA [39] from the transformed strains, 1× reaction Green GoTaq® Flexi buffer, 0.2 mM of each dNTP, 1.5 mM MgCl_2,_ 15 pmol of each primer and 0.125U of GoTaq® G2 Flexi DNA Polymerase (Promega, Fitchburg, U.S.A). The amplification program was: 2 min of initial denaturation at 95°C, 30 cycles of 60 s at 95°C, 45 s at 54°C, 10 s at 72°C or 205 s at 72°C (for Ca36pPHU and Ca36pPHUclpB, respectively), and a final extension of 5 min at 72°C.

### Analysis of *clpB*, *nodA* and *nodC* genes expression by semiquantitative RT-PCR

To confirm the expression of the extra-copy of the *clpB* gene cloned in pPHU231, a semiquantitative RT-PCR analysis was performed, as described in [40]. Total RNA of the wild-type strain (Ca36WT) and its derivates (strains Ca36pPHUclpB and Ca36pPHU) was extracted using the GeneJET™ RNA Purification Kit (ThermoFisher Scientific, Waltham, U.S.A), from bacteria grown in minimal medium [41] at 28°C for 24 hours, with a final OD_540_ of 0.4. DNA contamination was removed by DNase I (Roche Diagnostics, Basel, Switzerland) digestion, followed by RNA cleanup using GeneJET™ RNA Purification Kit. Approximately 250 ng of total RNA was subjected to reverse transcription for cDNA synthesis, using the RevertAid First Strand cDNA Synthesis kit (ThermoFisher Scientific, Waltham, U.S.A). Amplification of *clpB* gene was performed using the primers ClpBIntF 5’- CGCCGAACCAAGAACAATCC -3’ and CLPBIntR3 5’- GACCAGCGTGTGCATCTCATC -3’, which generate a fragment of 266 bp. This PCR reaction was performed in a final volume of 25 μL, using 3 μL of cDNA (diluted 50×), 1× reaction Green GoTaq® Flexi buffer, 0.2 mM of each dNTP, 1.5 mM MgCl_2,_ 15 pmol of each primer and 0.125U of GoTaq® G2 Flexi DNA Polymerase (Promega, Fitchburg, U.S.A). The amplification program was: 2 min of initial denaturation at 95°C, 30 cycles of 60 s at 95°C, 60 s at 56°C, 16 s at 72°C, and a final extension of 5 min at 72 C.

The analysis of the *nodA* and *nodC* genes expression was also performed in the *clpB*-transformed strain and in the Ca36pPHU strain. The total RNA of these strains was extracted from bacteria exposed to chickpea root exudates for 24h at 28°C, with a final OD_540_ of 0.4. The exudates were obtained as described in [42]. DNA contamination was removed as described previously and approximately 275 ng of total RNA was subjected to reverse transcription for cDNA synthesis. Amplification of the *nodA* gene was performed using the NodAIntF 5’- CCGAATGTCGAGTGGAAGTT -3’ and NodAIntR 5’- ctcgccaactttgatgaagc -3 primers, whereas the *nodC* gene was amplified using NodCIntF 5’- atggaccttctcaccacagc -3’ and NodCIntR 5’- tgtagcaggggatgatgaca -3 primers, generating fragments of 234bp and 201bp, respectively. The PCR reaction was performed in a final volume of 25 μL, using 2 μL of cDNA (diluted 100×), 1× reaction Green GoTaq® Flexi buffer, 0.2 mM of each dNTP, 1.5 mM MgCl_2,_ 15 pmol of each primer and 0.125U of GoTaq® G2 Flexi DNA Polymerase (Promega, Fitchburg, U.S.A). The amplification program was: 2 min of initial denaturation at 95°C, 30 cycles of 60 s at 95°C, 60 s at 54°C (*nodA*) or 56°C (*nodC*), 14 s (*nodA*) or 12 s (*nodC*) at 72°C, and a final extension of 5 min at 72°C.

The amplification of the 16S rRNA gene was used to normalize the relative *clpB*, *nodA* and *nodC* transcripts abundance, using primers IntF and IntR [43], which generate a fragment of 199 bp. The PCR reaction was performed to a final volume of 25 μL, using 1 μL of cDNA (diluted 100×), 1× reaction Green GoTaq® Flexi buffer, 0.2 mM of each dNTP, 1.5 mM MgCl_2,_ 15 pmol of each primer and 0.125U of GoTaq® G2 Flexi DNA Polymerase (Promega, Fitchburg, U.S.A). The amplification program was: 2 min of initial denaturation at 95°C, 30 cycles of 60 s at 95°C, 60 s at 56°C, 12 s at 72°C, and a final extension of 5 min at 72°C.

Densitometric analysis of ethidium bromide-stained agarose gels was performed using Kodak Digital Science 1D version 2.0.3 (Eastman Kodak Company, Rochester, U.S.A). Positive controls with total DNA of Ca36WT strain as template and negative controls using RNA without the addition of the reverse transcriptase enzyme were performed. Three biological replicates were performed for the expression analysis of the genes.

### Analysis of stress tolerance in free-living conditions

To characterize the phenotype of the Ca36pPHUclpB strain, namely its tolerance to stress conditions, bacterial growth was evaluated in liquid medium by measuring the OD_540_ every 24 h up to 168 h. Several heat and acidity stress conditions were tested and the control conditions were 28°C and pH7. For all treatments, bacterial cultures with an initial OD_540_ of 0.1 were prepared after an overnight growth, in Tryptone Yeast (TY) medium [44] for heat stress evaluation or in Yeast Extract Mannitol (YEM) broth, buffered as described in [45], for acid stress evaluation. The media were supplemented with tetracycline (10μg/mL) for the growth of the Ca36pPHU and Ca36pPHUclpB strains. Three replicas per treatment were performed.

To study the effect of heat stress, Ca36WT, Ca36pPHUclpB and Ca36pPHU strains were submitted to a continuous heat stress (37°C) and a heat shock (48°C during 30 min, followed by growth at 28°C). To evaluate the effect of acid stress, the bacterial cultures were submitted to acid shocks at pH5 and pH3 (during 1 h followed by growth at pH7).

### Evaluation of nodulation kinetics

To evaluate the nodulation kinetics, a hydroponic assay was conducted using chickpea plants inoculated with Ca36WT, Ca36pPHU or Ca36pPHUclpB strains. All the procedures were performed as described in [7]. Eight seeds per treatment were used and the number of nodules was evaluated every three days for a total of 24 days.

### Analysis of the symbiotic performance

In order to evaluate the symbiotic performance of the Ca36pPHUclpB strain, a plant growth assay was conducted in a growth chamber, under control (pH7) and stress conditions (pH5). Chickpea seeds (variety ELIXIR, cultivar CHK 3236) were surface-sterilized and pregerminated as described previously [46]. After germination, the seeds were transferred to plastic pots filled with sterile vermiculite and subsequently inoculated with the Ca36WT, Ca36pPHU or Ca36pPHUclpB strains. These strains were previously grown overnight in TY medium at 28°C (~18 h). The cell suspension was centrifuged at 8000g during 5 min and resuspended in fresh TY medium. The OD_540_ was adjusted at 1.0, and 1 ml of the bacterial suspension was used to inoculate each seedling. Five replicates were used for each treatment. Chickpea plants were grown for 8 weeks as previously described [46].

Plants were watered, three times per week, with 100 mL of a nitrogen-free nutrient solution [47], with its pH adjusted to 7 (control) or 5 (stress) as previously described [48]. Uninoculated plants watered with a nutrient solution containing 0.1% of KNO_3_ (as nitrogen source) were used as positive controls. Uninoculated plants watered with a nitrogen-free nutrient solution were used as negative controls. After 8 weeks, the plants were harvested and several parameters were measured, such as number of nodules (NN), nodule dry weight (NDW), shoot dry weight (SDW) and root dry weight (RDW). The average weight per nodule (AWN) was calculated as a ratio between NDW and NN. Symbiotic Effectiveness (SE) was determined using both positive and negative controls, as described in [49].

To confirm the presence of the pPHU231 or pPHUclpB plasmids in nodules formed by the transformed strains, recovery of bacteria from the nodules was conducted, following the procedures described in [35].

### Analysis of root hair curling

For the analysis of the chickpea root hair curling induced by the Ca36pPHUclpB, Ca36pPHU and wild-type strains, 2-day-old germinated chickpea seeds were inoculated as previously described [50]. The seedlings were inoculated with a bacterial suspension with an OD_540_ of 0.4, after an overnight growth on YEM broth. A minimum of 16 seedlings per strain were analysed. The differentiation zone of the roots was observed under an Olympus BX41 microscope, and images were captured using an Olympus SC30 digital camera with analySIS getIT software (version 5.2).

### Analysis of nodules histology

Roots and nodules were excised from 8-week-old plants and processed for light microscopy using Bright field and phase-contrast optics. The internal morphological features of chickpea nodules were examined by microscopy after Toluidine blue staining. Nodules were fixed in 4% formaldehyde in 50 mM phosphate buffer (pH 8), dehydrated in an increasing ethanol series, and embedded in paraffin. Toluidine blue-stained sections (2 μm) of embedded nodules were examined by light microscopy under a Nikon SMZ800 stereomicroscope and Nikon eclipse 80i microscope. The images were captured using a Nikon DS-Fi1 and Nikon DS-Ri1 camera respectively.

### Statistical analysis

The data obtained from RT-PCR analyses and from the chickpea plant-growth assays were analyzed using one-way ANOVA (*P* < 0·05). The post hoc Tukey test was used to compare the means and indicate which treatments have significant differences. Statistical analysis was carried out using SPSS V.21 software (SPSS Inc., Chicago, U.S.A).

## Results

### Confirmation of bacterial transformation and analysis of *clpB* gene expression in the *clpB*-transformed strain

To confirm the bacterial transformation with the pPHU231 or pPHUclpB plasmids, a PCR amplification using M13 primers was performed. As expected, a band of ~4500bp (3059bp corresponding to the *clpB* gene including its promoter and terminator regions, plus ~1500bp of plasmid sequence) was obtained using as template the total DNA of the Ca36pPHUclpB strain. A band with ~1500bp was obtained using as template the total DNA of the Ca36pPHU strain, confirming the presence of the expression vector (data not shown).

**Fig 1:**
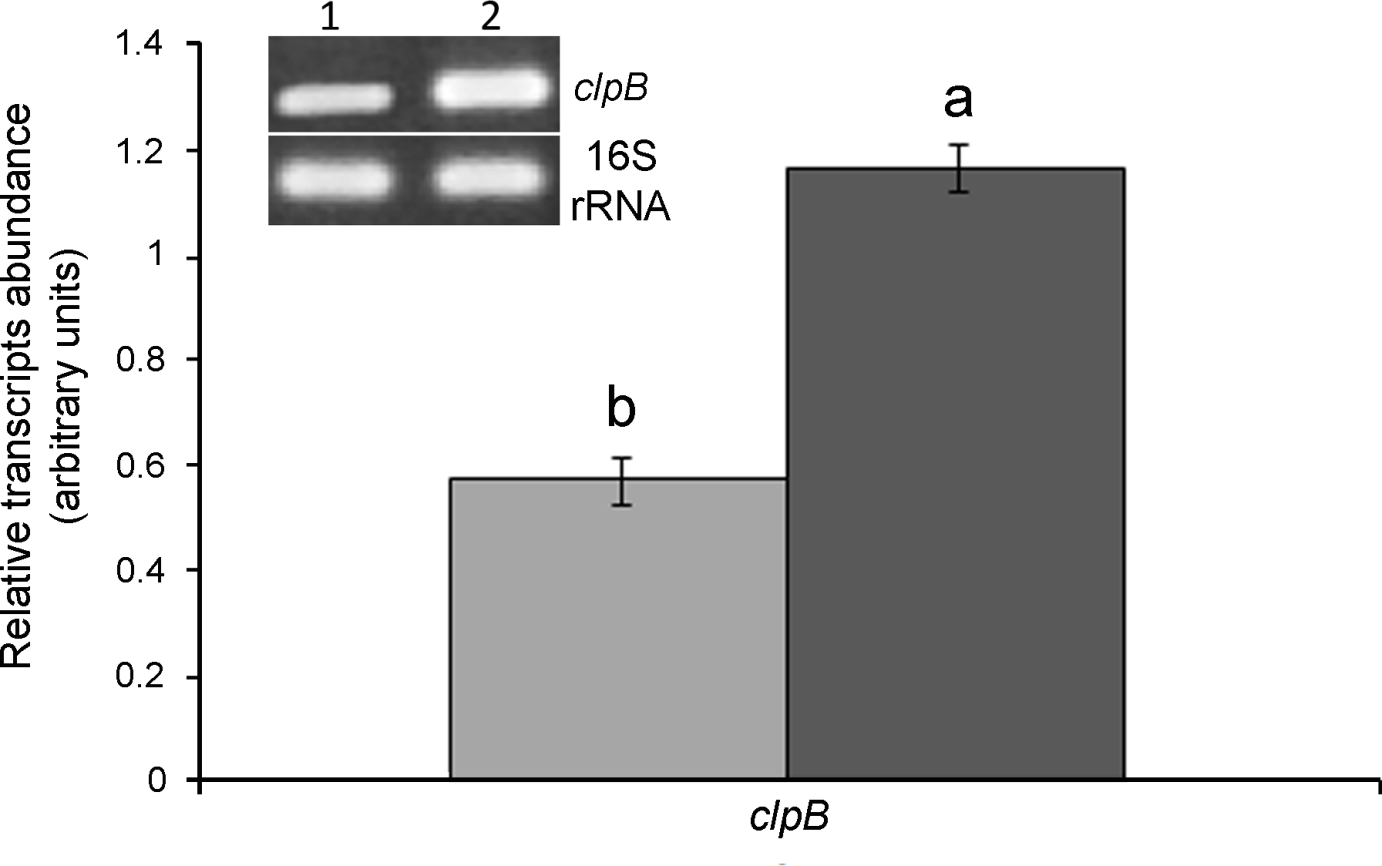
Analysis of *clpB* gene transcription by semiquantitative RT-PCR in *M*. *mediterraneum* Ca36pPHU (lane 1, light gray bar) and Ca36pPHUclpB (lane 2, dark gray bar) strains. The relative *clpB* transcript abundance was normalized against the amplification of a fragment of 16S rRNA gene. For the quantification of RT-PCR analysis, data are presented as the mean and standard error values of three independent biological replicates. Different letters (a, b) correspond to statistical significant differences (*P* < 0.05).

To confirm that extra-copies of the *clpB* gene in the Ca36pPHUclpB strain were being transcribed, the *clpB* transcript abundance was evaluated by semiquantitative RT-PCR, and compared with the levels in Ca36pPHU strain. In the Ca36pPHUclpB strain the transcriptional levels of the *clpB* gene were ~2 fold higher than those detected in Ca36pPHU strain. (Fig 1). The Ca36WT and Ca36pPHU strains presented similar *clpB* expression levels (data not shown).

### Evaluation of the stress tolerance of the wild-type and transformed strains in free-living conditions

**Fig 2:**
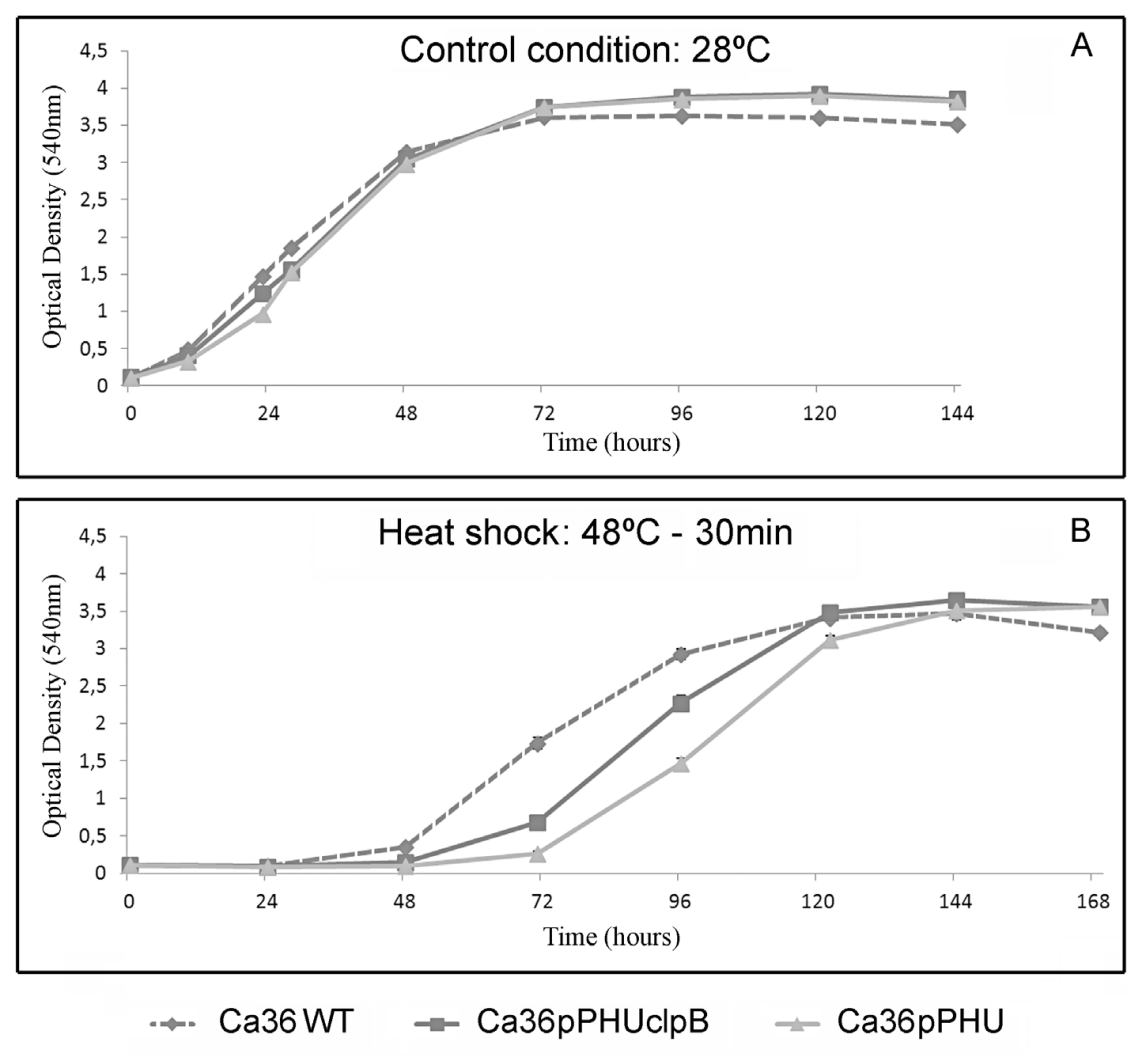
Growth curves of *M*. *mediterraneum* UPMCa36^T^ wild-type strain (Ca36WT) and its derivatives (Ca36pPHU and Ca36pPHUclpB) under control and heat shock conditions. Bacterial growth at 28°C (A). Bacterial growth after a heat shock of 48°C during 30 min, followed by growth at 28°C (B).

To determine the effects of extra-copies of the *clpB* gene in *M*. *mediterraneum* UPM-Ca36^T^ stress response, the growth of the Ca36WT, Ca36pPHU and Ca36pPHUclpB strains was evaluated in liquid medium under control and stress conditions (Fig 2).

At control conditions (28°C), both transformed strains showed a similar growth curve (Fig 2A). However, when these bacteria were submitted to a heat shock (48°C, 30 min), a higher growth rate in the exponential phase was observed for the Ca36pPHUclpB strain in comparison with the Ca36pPHU (Fig 2B). This suggests the involvement of the ClpB chaperone protein in the recovery from heat shock. The lower growth rate of the Ca36pPHU and Ca36pPHUclpB strains in comparison to the Ca36WT strain, can be justified by the energetic cost of maintaining the plasmid, which becomes more significant under stressful conditions.

Both transformed strains, Ca36pPHU and Ca36pPHUclpB, showed a similar growth curve after an acid shock at pH5 and pH3 for 1h (data not shown). Under continuous heat (37°C) a very low growth rate was observed for the wild-type and both transformed strains (data not shown).

### Nodulation kinetics of the wild-type and transformed strains

**Fig 3:**
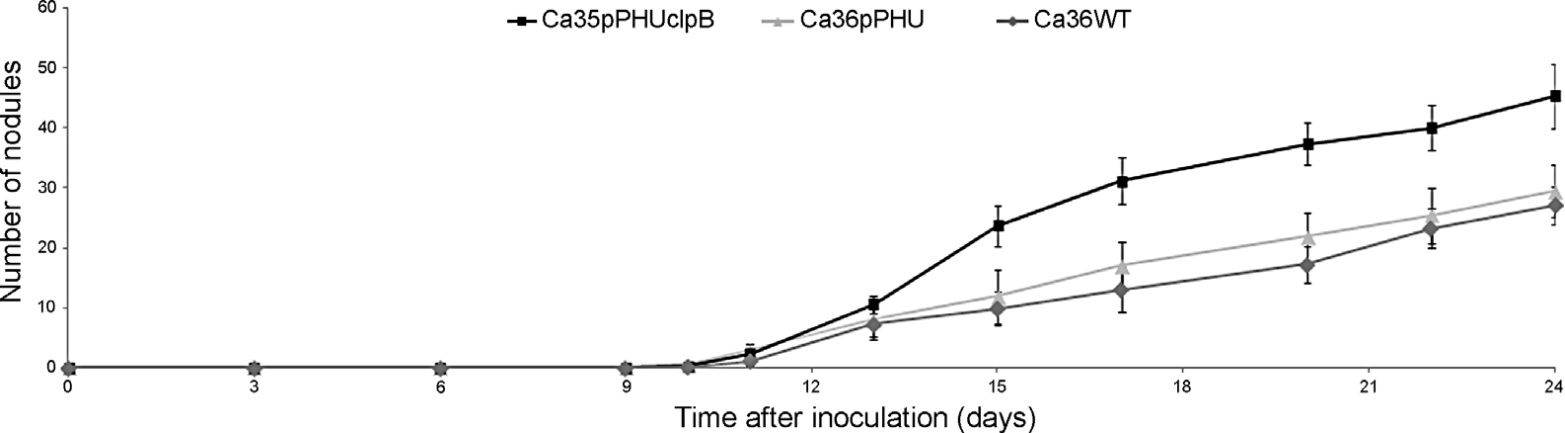
Nodulation kinetics of chickpea plants inoculated with Ca36WT, Ca36pPHU or Ca36pPHUclpB strains during 24 days after inoculation. Each point represents the mean and standard error values of eight plants per treatment.

In order to evaluate the effect of the extra-*clpB* gene copies in the initial processes of the symbiotic relationship, a hydroponic plant assay was conducted to compare the nodulation kinetics in plants inoculated with the transformed and the wild-type strains. The nodulation kinetics analysis shows that 10 days after inoculation the chickpea plants inoculated with Ca36pPHUclpB, Ca36pPHU and Ca36WT strains display the first nodules (Fig 3). However, the rate of nodulation is higher with the Ca36pPHUclpB strain. At 17 days after inoculation and onwards, a significantly higher number of nodules (NN) was observed in plants inoculated with the Ca36pPHUclpB strain, compared to the plants inoculated with the Ca36WT and Ca36pPHU strains. No significant differences were observed in the nodulation kinetics between the Ca36WT and Ca36pPHU strains (Fig 3).

### Evaluation of symbiotic performance of the wild-type and transformed strains

**Fig 4:**
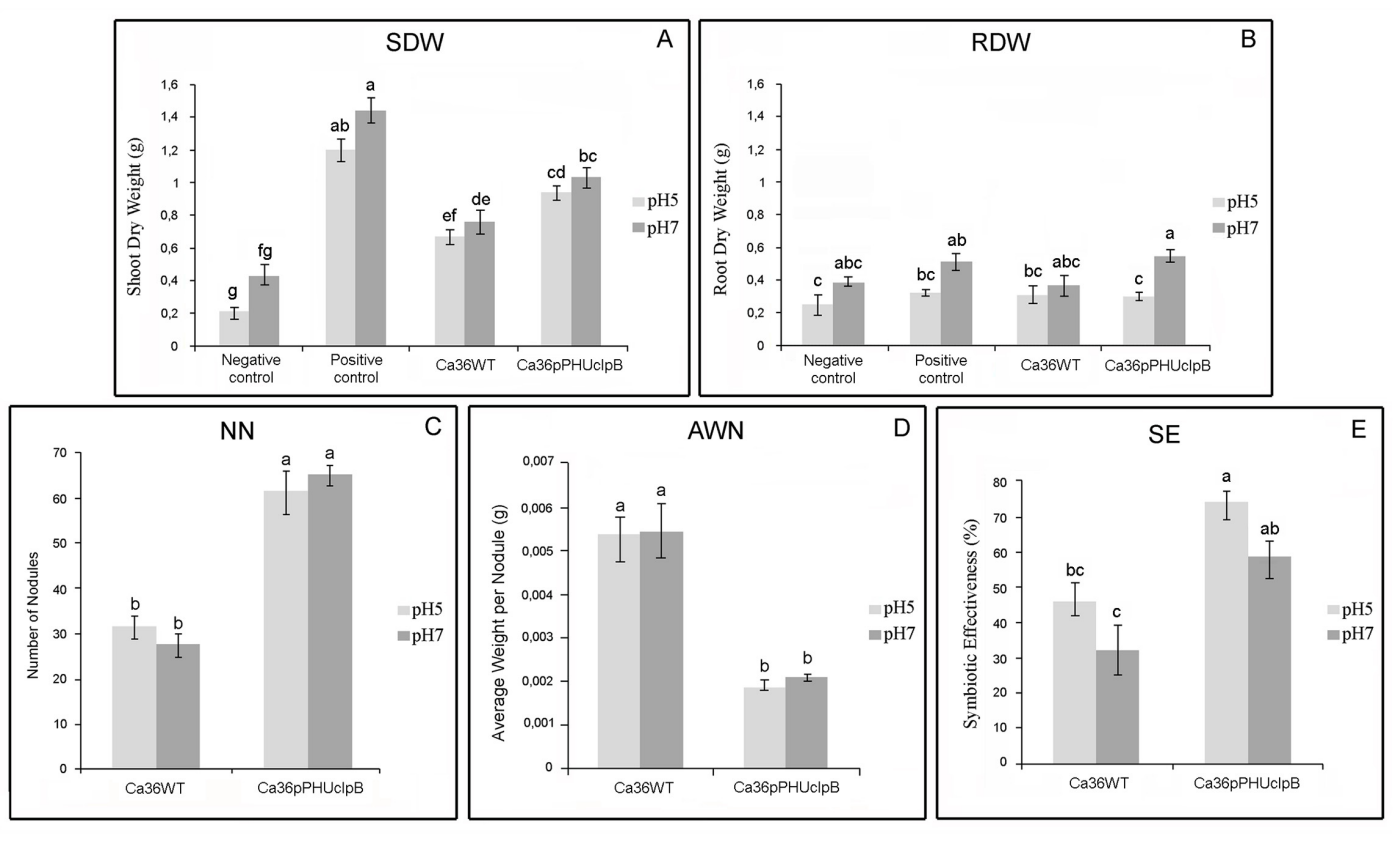
Results obtained from a plant growth assay performed under control (pH7) and stress conditions (pH5). The chickpea plants were inoculated with the *M*. *mediterraneum* UPM-Ca36^T^ wild-type strain (Ca36WT) and Ca36pPHUclpB. SDW—Shoot dry weight (A). RDW—Root dry weight (B). NN—Number of Nodules (C). AWN—Average Weight per Nodule (D). SE-Symbiotic Effectiveness (E). Data correspond to the mean and standard error of five plant replicates (n = 5) per treatment. Different letters (a-g) correspond to statistical significant differences (*P<0*.*05*). Dark grey bars correspond to results obtained under control conditions (pH7). Light grey bars correspond to results obtained in plants subjected to pH stress conditions (pH5).

The symbiotic performance of the wild-type and transformed strains was evaluated under control conditions (pH7) and acid stress (pH5). No significant differences were obtained between the symbiotic performance of Ca36WT and Ca36pPHU strains (data not shown), therefore this section will only present the results referring to the Ca36WT and the Ca36pPHUclpB strains (Fig 4).

As expected, the acid conditions negatively affected the chickpea growth, since the positive and negative control plants show values of shoot dry weight (SDW) and root dry weight (RDW) lower at pH5 compared to pH7 (Fig 4A and 4B). Minor differences between the parameters obtained at pH5 and pH7 were observed in inoculated plants, either with the Ca36WT or with the Ca36pPHUclpB strain (except for RDW with Ca36pPHUclpB strain that shows a significantly higher value at pH7 than at pH5, Fig 4B). These results suggest that rhizobia inoculation *per se* can alleviate the negative effect of low pH in chickpea plants.

Plants inoculated with Ca36pPHUclpB strain showed a significant increase in the SDW and NN, compared to the plants inoculated with the Ca36WT strain, at both pH conditions tested (Fig 4A and 4C). The SDW of chickpea plants inoculated with the Ca36pPHUclpB was 41% (at pH5) and 36% (at pH 7) higher than those inoculated with the Ca36WT. The remarkable increase in the NN was around 95% at pH5 and 137% at pH7. More importantly, the symbiotic effectiveness (SE) of Ca36pPHUclpB strain was ~60% and 83% higher compared to the wild-type strain, at pH5 and pH7 conditions, respectively (Fig 4E). These results indicate that the extra-copies of the *clpB* gene improved the symbiotic performance of the *M*. *mediterraneum* UPM-Ca36^T^ strain.

Despite the increase of NN found in plants inoculated with the Ca36pPHUclpB, the average weight per nodule (AWN) was significantly lower than the one obtained in plants inoculated with Ca36WT strain, since the nodules resulting from the Ca36pPHUclpB present a smaller size (Fig 4D). This suggests that the expression of extra-copies of the *clpB* gene in *M*. *mediterraneum* UPM-Ca36^T^ contributes not only to an increase in the NN but also to changes in the nodule development. The nodule dry weight (NDW) of plants inoculated with both strains is not significantly different (data not shown).

### Analysis of the chickpea root hair curling induced by the wild-type and transformed strains

**Fig 5:**
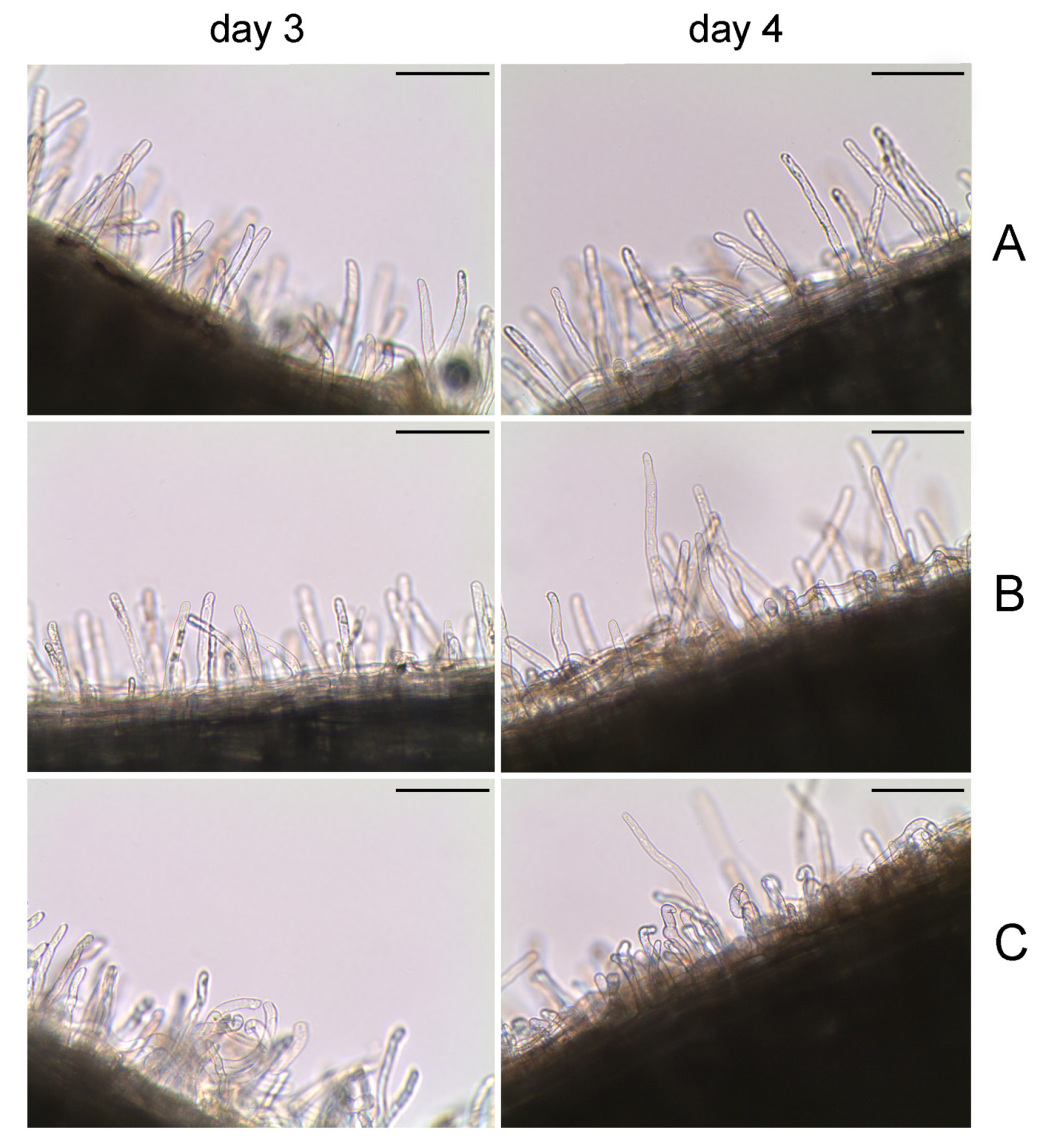
Microscopic analysis of root hair curling of chickpea plants inoculated with Ca36WT, Ca36pPHU or Ca36pPHUclpB. This analysis was performed in the third and fourth days after inoculation. Plants inoculated with the Ca36WT strain (A). Plants inoculated with the Ca36pPHU strain (B). Plants inoculated with the Ca36pPHUclpB strain (C). Scale bar: 0.053 μm.

In order to evaluate if the higher NN produced by the *clpB*-transformed strain, could be associated with differences in the root hair curling, this process was monitored for several days in seedlings inoculated with the wild type and the transformed strains. At day 3, a more evident root hair curling was observed in seedlings inoculated with the strain Ca36pPHUclpB. At day 4, the seedlings inoculated with either of the three strains showed root hair curling, however, the *clpB*-transformed strain induced a higher number of curled root hairs in comparison with the Ca36WT and Ca36pPHU strains (Fig 5). The Ca36WT and Ca36pPHU strains present a similar ability in inducing the root hair curling (Fig 5). These results suggest a higher induction of root hair curling by the *clpB*-transformed strain, which probably contributes to the higher nodulation rate obtained with this strain in the plant trials (Fig 3 and Fig 4).

### Histological analysis of nodules

**Fig 6:**
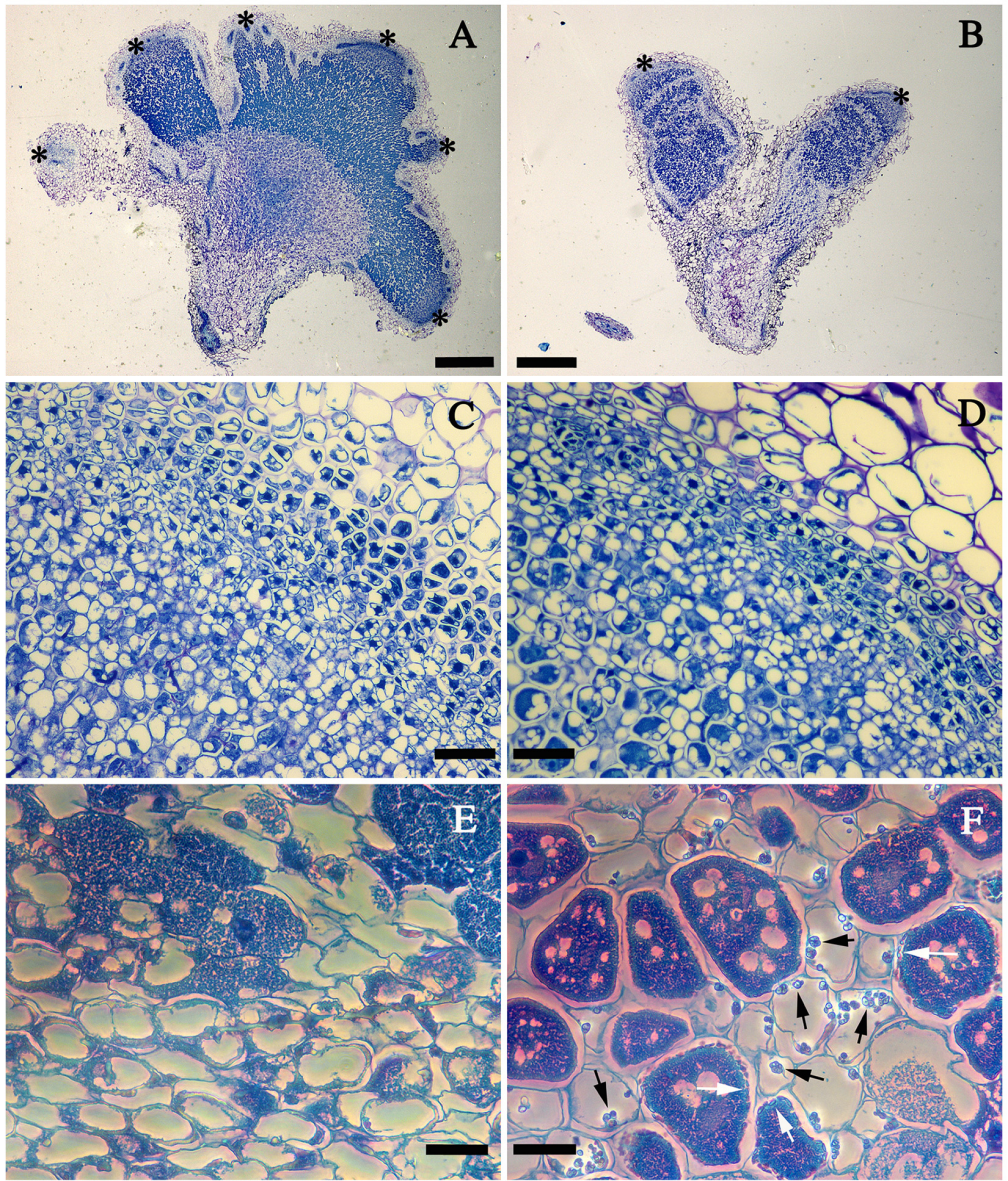
Nodule development in chickpea. Portions of nodulated roots inoculated with the Ca36pPHU (A), wild-type Ca36WT (C, E), or Ca36pPHUclpB strains (B, D, F) are shown. A and B, Stereophotomicrographs of sections of embedded nodules stained with Toluidine Blue. C to F, Photomicrographs of embedded nodules stained with Toluidine Blue. A to D, bright field light microscopy. E and F, Phase contrast microscopy. A and B, whole nodules (asterisks indicate meristematic zones). C and D, meristematic zones at higher magnification. E and F, infection zones (Black arrows indicate particles inside uninfected cells; White arrows indicate particles inside infected cells). Scale Bars: 400 μm (A and B); 100 μm (C and D); 50 μm (E and F).

To further define the effect of *clpB*-transformed strain on symbiotic development, we examined embedded nodule sections induced by Ca36WT, Ca36pPHU and Ca36pPHUclpB strains. No differences were obtained between the nodule cytology of Ca36WT and Ca36pPHU strains. Bright field light microscopy (Fig 6A–6D) showed that all nodules examined had the typical histology of indeterminate effective nodules with differentiated meristematic, infection, and bacteroid zones (Fig 6A and 6B). However, nodules induced by Ca36WT or Ca36pPHU (Fig 6A) were greater in size and have a higher number of meristematic zones (asterisks in Fig 6A) than nodules induced by CapPHUclpB (asterisks in Fig 6B). Amplification of these meristematic zones (Fig 6C and 6D) showed no difference between all nodules analyzed. Phase contrast microscopy (Fig 6E and 6F) revealed the presence of particles only in the nodules induced by Ca36pPHUclpB (arrows in Fig 6F). These particles were observed mainly in the infection zone inside of both infected (Fig 6F, white arrows) and uninfected cells (Fig 6F, black arrows). The appearance of these particles differs according to the type of cells. In infected cells resemble vesicles (Fig 6F, white arrows) and in uninfected cells they are more similar to granules (Fig 6F, black arrows). However, we must proceed to further studies to confirm the nature of these particles and their involvement in symbiotic performance.

### Analysis of *nodA* and *nodC* genes expression in the *clpB*-transformed strain

**Fig 7:**
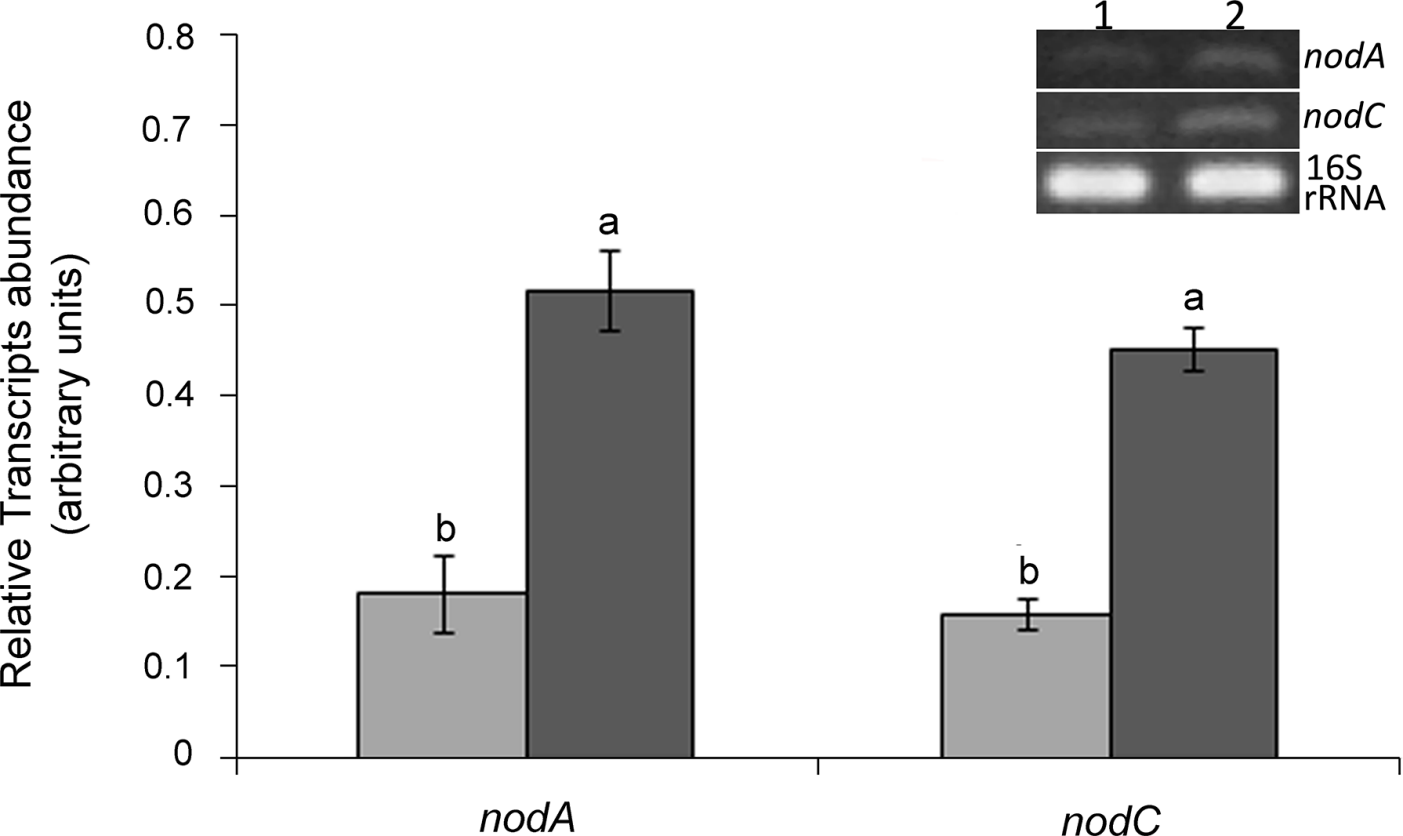
Analysis of *nodA* and *nodC* gene transcription by semiquantitative RT-PCR in the Ca36pPHU (lane 1, light gray bars) and *clpB*-transformed (lane 2, dark gray bars) strains. To normalize the relative *nodA* and *nodC* transcripts abundance the amplification of a fragment of 16S rRNA gene was also performed. Data in the graph correspond to the mean and standard error values of three independent biological replicates. Different letters (a, b) correspond to statistical significant differences (*P* < 0.05).

To understand how the *clpB* extra-copies could improve the symbiotic performance of the strain, namely its nodulation efficiency, the expression of the nodulation genes *nodA* and *nodC* was evaluated by semiquantitative RT-PCR (Fig 7). The transcriptional levels of both *nodA* and *nodC* genes in the *clpB*-transformed strain were ~3 folds higher than those detected for the Ca36pPHU strain, suggesting higher levels of Nod factors in Ca36pPHUclpB. This could contribute to the enhanced induction of root hair curling and nodulation efficiency in the plants inoculated with the Ca36pPHUclpB strain.

## Discussion

The improvement of rhizobia stress tolerance may allow the development of more efficient inoculant strains to be used in fields affected by environmental stress conditions. The modification of rhizobia by transformation with chaperone genes, coding for proteins commonly involved in stress response, could act in two beneficial ways: improvement of stress tolerance and symbiotic effectiveness (SE). Several chaperones were found to be directly involved in the symbiotic process (e.g. [7, 8]). Our previous results clearly showed the involvement of ClpB chaperone in plant-rhizobia nodule formation and development: a *clpB* knockout mutant of *M*. *ciceri* LMS-1 displays a delay in the nodulation of chickpea plants, as well as a lower number of bacteroids, when compared to the wild-type [7]. Therefore, with the major goal of improving the stress tolerance and symbiotic performance of *M*. *mediterraneum* UPM-Ca36^T^, this strain was genetically transformed with an extra-copy of the *clpB* gene cloned in the expression vector pPHU231.

### Role of *clpB* in bacterial stress response

Different reports point towards an important role of ClpB in bacterial heat shock tolerance, namely in *E*. *coli* [16], *Brucella suis* [22], *Vibrio cholera* [51], as well as in rhizobia, as is the case of *Ensifer meliloti* [52], *M*. *ciceri* [7] and *M*. *loti* [53]. In the present work, the evaluation of growth under abiotic stress conditions showed that the expression of additional copies of the *clpB* gene led to a higher growth rate after a heat shock, indicating that ClpB contributes to the heat shock response in *M*. *mediterraneum* UPM-Ca36^T^. This is in agreement with the reported overexpression of the *clpB* gene of *M*. *loti* MAFF303099 [53], as well as the overproduction of this chaperone in a *M*. *ciceri* strain [7], in response to heat shock. The fact that the transcription of the *clpB* extra-copies is under the control of the native promoter suggests that the native- and extra-copy genes are similarly regulated. Therefore, it is expected that the phenotypic effects due to the extra-copies will be more pronounced in stress conditions that significantly upregulate the native *clpB*, such as heat shock.

Regarding the tolerance to acid shock in free living conditions, no differences were observed between the growth curves of Ca36pPHUclpB and Ca36pPHU strains. These results suggest that the presence of *clpB* extra-copies is insufficient to overcome the negative effect of acid stress in *M*. *mediterraneum* UPM-Ca36^T^, which is very sensitive to this condition. In *Mesorhizobium*, the role of ClpB in acid stress tolerance is not yet clear. In *M*. *ciceri* LMS-1, the involvement of ClpB in acid stress response was only detected in cells submitted to very severe acid conditions [7]. In a transcriptome analysis of *M*. *loti* MAFF303099 submitted to an acidic shock, a very slight underexpression of *clpB* gene was detected [54]. Therefore, more studies are required in order to clarify the ClpB involvement in *Mesorhizobium* tolerance to acidity.

### Role of *clpB* in the symbiotic relationship legume-rhizobia

With the main purpose of evaluating the effects of *clpB* extra-copies in the symbiotic performance of *M*. *mediterraneum* UPM-Ca36^T^, plant growth trials were performed (hydroponic assay for nodulation kinetics and pot assay for symbiotic effectiveness).

Since acidity is a common problem in the fields, the pot assay was carried out under control (pH7) and acid stress conditions (pH5). In both pH conditions, the presence of *clpB* extra-copies showed a beneficial effect in the symbiotic performance of this mesorhizobium strain, with a significant increase of approximately 60% and 83% in the SE, at pH5 and pH7, respectively. The NN and SDW of plants inoculated with the Ca36pPHUclpB strain were significantly higher than the ones obtained with Ca36WT. Similarly, the root hair curling observed in plants inoculated with Ca36pPHUclpB was higher than the one obtained with Ca36WT. These results support the role of the *clpB* extra-copies in the symbiotic performance, probably through the increase in root hair curling and nodulation abilities, and leading to the development of more efficient nodules. Histological analysis shows that the nodules induced by Ca36pPHUclpB present some particles that are not observed in the nodules induced by the Ca36WT or Ca36pPHU strains. However, if and how these particles contributed to the higher symbiotic performance of the Ca36pPHUclpB remains unclear. Futher studies are required to confirm the nature of these particles and their involvement in symbiotic performance. The higher induction of root hair curling observed in seedlings inoculated with the *clpB*-transformed strain probably leads to the higher rate of nodulation. Overall, these results suggest that the extra-copies of the *clpB* gene promotes, at least, the nodulation process, which contributes to an increased symbiotic effectiveness.

Transcriptomic and proteomic analyses of bacteroids suggest that chaperone genes are involved in the symbiotic process [55–58]. For instance, studies with the major chaperone GroEL have shown that it modulates NodD activity, which in turns regulates the *nod* genes expression in *E*. *meliloti*. *E*. *meliloti groEL1* mutants presented a reduction in the *nod* gene expression and formed ineffective nodules in different legumes [6]. More recently, Brígido et al. [7] clearly demonstrated that the chaperone ClpB is involved in the mesorhizobium-chickpea nodulation process. The delay in nodule formation by a *clpB* mutant was suggested to be related to its inability to properly activate the expression of the *nod* genes, probably due to an inappropriate folding of the NodD protein [7].

Agreeing with previous studies, our data reinforce the importance of ClpB in the symbiosis, namely in the nodulation process and ultimately in the SE of the strain. Thus, one possible explanation for the influence of the *clpB* extra-copies in the SE is the involvement of ClpB in the disaggregation and folding of proteins related to the symbiosis, namely nodulation proteins, such as NodD. This is the major activator of *nod* genes that encode the enzymes involved in the synthesis and secretion of Nod factors [59], which are perceived by the host plant and trigger the root hair curling [60]. The hypothesis that extra ClpB chaperone disaggregates and folds symbiosis-related proteins, such as the *nod* activator NodD, is supported by our RT-PCR data showing an increase of ~3 fold in the expression of the nodulation genes *nodA* and *nodC* in the *clpB*-transformed strain, compared to the Ca36pPHU strain. The increased expression of nodulation genes detected in the *clpB*-transformed strain suggests that this strain synthesizes higher levels of Nod factors. This is supported by the observation of more curled root hairs, as well as a higher NN, in plants inoculated with the *clpB*-transformed strain, compared to plants inoculated with Ca36pPHU or wild type strains.

It has been suggested that rhizobia encounter stress conditions when entering or within the cells of the plant hosts [61], such as acidity or microaerobiosis, requiring the action of several molecular chaperones and proteases. In fact, several studies in rhizobia showed the induction of chaperone genes, such as *clpB*, under acidity or microaerobic conditions, supporting their involvement in the protection of proteins from denaturation and aggregation within the host cells [23, 62, 63]. Despite the fact that in free-living conditions, no increase in acid stress tolerance was obtained with the *clpB*-extra copies, it is possible that in the rhizosphere and root nodules the conditions are more complex involving a combination of different stresses. This could explain the beneficial effect of the *clpB*-extra copies in overcoming stress and ultimately increasing the symbiotic effectiveness.

## Conclusions

Overall, our data show that the transformation of the *M*. *mediterraneum* UPM-Ca36^T^ strain with extra-copies of the *clpB* gene has a clear beneficial effect in its symbiotic performance. It is probable that the contribution of the extra-copies of the *clpB* in the chickpea-mesorhizobium symbiosis may be related to a more efficient disaggregation of proteins involved in the symbiosis, particularly in the molecular signaling between the two partners. In addition, the extra-copies of the *clpB* may confer a higher ability to counteract the stress conditions found in the rhizosphere and within the root nodule.

The present work shows that it is possible to improve the symbiotic effectiveness, as well as the stress tolerance (namely to heat shock) of *M*. *mediterraneum* UPM-Ca36^T^, by overexpressing the *clpB* chaperone gene. These results have potential applications, namely in obtaining more efficient inoculants for field crops, particularly under environmental stress conditions. Future studies could involve the integration of an extra-copy of *clpB* gene in the chromosome of the *M*. *mediterraneum* UPM-Ca36^T^, in order to increase its stability in the bacterial genome.

Although our previous report [7] together with the present study contribute to the elucidation of the involvement of the chaperone ClpB in the symbiosis rhizobia-chickpea, the molecular mechanisms behind its role in the symbiosis are not fully characterized, requiring further studies. This report describes the first successful improvement of the SE of a rhizobium by its transformation with an extra copy of a chaperone gene.
